# Effects of immunophilin inhibitors and non-immunosuppressive analogs on coronavirus replication in human infection models

**DOI:** 10.3389/fcimb.2022.958634

**Published:** 2022-09-21

**Authors:** Emilia J. Berthold, Yue Ma-Lauer, Ashesh Chakraborty, Brigitte von Brunn, Anne Hilgendorff, Rudolf Hatz, Jürgen Behr, Felix Hausch, Claudia A. Staab-Weijnitz, Albrecht von Brunn

**Affiliations:** ^1^ Institute of Lung Health and Immunity and Comprehensive Pneumology Center with the Comprehensive Pneumology Center Munich (CPC-M) bioArchive, Helmholtz-Zentrum München, Munich, Germany; ^2^ Max von Pettenkofer Institute, Department of Virology, Faculty of Medicine, Ludwig-Maximilians-Universität (LMU), Munich, Germany; ^3^ German Center for Infection Research, Munich, Germany; ^4^ Thoraxchirurgisches Zentrum, Klinik für Allgemeine, Viszeral-, Transplantations-, Gefäß- und Thoraxchirurgie, Klinikum Großhadern, Ludwig-Maximilians-Universität, Munich, Germany; ^5^ Medizinische Klinik und Poliklinik V, Klinikum der Ludwig-Maximilians-Universität (LMU), Munich, Germany; ^6^ Department of Chemistry and Biochemistry, Technical University Darmstadt, Darmstadt, Germany

**Keywords:** HCoV-229E, Cyclosporin A, FK506, non-immunosuppressive analogs, pHBECs, tacrolimus

## Abstract

**Rationale:**

Human coronaviruses (HCoVs) seriously affect human health by causing respiratory diseases ranging from common colds to severe acute respiratory diseases. Immunophilins, including peptidyl-prolyl isomerases of the FK506-binding protein (FKBP) and the cyclophilin family, are promising targets for pharmaceutical inhibition of coronavirus replication, but cell-type specific effects have not been elucidated. FKBPs and cyclophilins bind the immunosuppressive drugs FK506 and cyclosporine A (CsA), respectively.

**Methods:**

Primary human bronchial epithelial cells (phBECs) were treated with CsA, Alisporivir (ALV), FK506, and FK506-derived non-immunosuppressive analogs and infected with HCoV-229E. RNA and protein were assessed by RT-qPCR and immunoblot analysis. Treatment with the same compounds was performed in hepatoma cells (Huh-7.5) infected with HCoV-229E expressing *Renilla* luciferase (HCoV-229E-RLuc) and the kidney cell line HEK293 transfected with a SARS-CoV-1 replicon expressing *Renilla* luciferase (SARS-CoV-1-RLuc), followed by quantification of luminescence as a measure of viral replication.

**Results:**

Both CsA and ALV robustly inhibited viral replication in all models; both compounds decreased HCoV-229E RNA in phBECs and reduced luminescence in HCoV-229E-RLuc-infected Huh7.5 and SARS-CoV-1-RLuc replicon-transfected HEK293. In contrast, FK506 showed inconsistent and less pronounced effects in phBECs while strongly affecting coronavirus replication in Huh-7.5 and HEK293. Two non-immunosuppressive FK506 analogs had no antiviral effect in any infection model.

**Conclusion:**

The immunophilin inhibitors CsA and ALV display robust anti-coronaviral properties in multiple infection models, including phBECs, reflecting a primary site of HCoV infection. In contrast, FK506 displayed cell-type specific effects, strongly affecting CoV replication in Huh7.5 and HEK293, but inconsistently and less pronounced in phBECs.

## 1 Introduction

Coronaviruses (CoVs) represent a diverse group of pathogens (Hu et al., 2021), which includes seven members that cause respiratory tract diseases in humans. HCoV-229E, -NL63, -OC43, and HKU-1 are mildly pathogenic and cause seasonal common cold symptoms. In contrast, MERS-CoV, SARS-CoV-1, and the newly emerged SARS-CoV-2 can cause severe infections of the respiratory tract that may ultimately lead to lung failure and death. The COVID-19 pandemic has exposed a lack of therapeutic options to treat coronavirus-induced disease, posing a significant threat to human health and global economic, political, and social stability. To this date, the COVID-19 pandemic alone has caused more than half a billion confirmed cases, including more than six million deaths worldwide (World Health Organization, 2022). The high probability of coronavirus evolution, zoonotic spillover, and thus novel CoV strains or variants of concern emerging in the future further emphasizes the need for broadly applicable pan-coronavirus inhibitors.

Immunophilins are protein folding catalysts with *cis-trans* peptidyl prolyl isomerase activity. They comprise two protein families: cyclophilins and FK506-binding proteins (FKBPs). Both FKBPs and cyclophilins have previously been shown to directly interact with various coronaviral proteins (Pfefferle et al., 2011; Carbajo-Lozoya et al., 2012; Gordon et al., 2020), suggesting that immunophilins are important host factors for coronavirus replication and thus promising targets for pharmaceutical inhibition. They are also involved in protein folding as molecular chaperones and play a role in receptor signaling and protein trafficking. They are often cell type-specifically expressed and act on specific folding substrates (Lammers et al., 2010; Staab-Weijnitz et al., 2015; Preisendorfer et al., 2022).

As the name suggests, many immunophilins bind immunosuppressive drugs in the PPI active site, thus inhibiting PPI activity. FKBPs, e.g., FKBP12 (gene name *FKBP1B*) bind to FK506 (tacrolimus), while cyclophilins bind cyclosporine A (CsA). When bound to cyclophilin A (CypA, gene name *PPIA*) and FKBP12, respectively, CsA and FK506 are well-established calcineurin inhibitors, which inhibit T-cell activation and thus display strong immunosuppressive effects.

Previous studies have shown that CsA, the non-immunosuppressive CsA analog Alisporivir (ALV), and FK506 inhibit coronavirus replication largely independent of virus strain (de Wilde et al., 2011; Carbajo-Lozoya et al., 2012; Sauerhering et al., 2020; Dittmar et al., 2021; Sauerhering et al., 2022). Except for the study by Sauerhering et al. (2022), which did not explore FK506 analogs, these studies were largely based on human cell lines frequently employed in virology research, like Huh-7.5 (a hepatoma cell line) and CaCo-2 cells (a colorectal adenocarcinoma cell line). However, coronaviruses primarily infect airway epithelial cells (Lukassen et al., 2020; Ziegler et al., 2020), underscoring the need for studies in more relevant *in vitro* models like, e.g., primary human bronchial epithelial cells (phBECs) cultured at the air-liquid interface (ALI).

Here, we initially set out to assess whether CsA and FK506 inhibit coronavirus replication in phBECs. To this end, we infected phBECs with HCoV-229E using two experimental strategies, i.e., pretreatment with an inhibitor or simultaneous treatment with infection. As the strong immunosuppressive properties of both drugs may exacerbate an infection and lead to a worse clinical outcome, we included the non-immunosuppressive compounds ALV (CsA analog), 16j (Pomplun et al., 2018)) and 19^(S)-Me^ (Bauder et al., 2021)) (FK506 analogs) in our study. While CsA and ALV consistently inhibited viral replication, FK506 and its analogs showed variable or no effects, respectively, on phBECs. This was in contrast to other human infection models like Huh-7.5 and HEK293, where not only CsA and ALV but also FK506 inhibited coronaviral replication, suggesting cell type-specific inhibition of coronaviral replication by FK506.

## 2. Materials and methods

### 2.1 Patient material

Primary human bronchial epithelial cells (phBECs) of non-CLD (Chronic Lung Disease) were obtained from the CPC-M bioArchive at the Comprehensive Pneumology Center (CPC, three donors). All phBECs were from patients undergoing lung tumor resections and were isolated from histologically normal regions adjacent to the resected lung tumors. Isolation and expansion of phBECs were performed as described previously (Mastalerz et al., 2022). The study was approved by the local ethics committee of the Ludwig-Maximilians University of Munich, Germany (Ethic vote #19-630). Written informed consent was obtained from all study participants.

### 2.2 Compounds

ALV was provided by Novartis (Basel, Switzerland) and used in phBECs at 10 µM and in Huh-7.5 and HEK293 cells up to 30 µM. CsA was obtained from Sigma-Aldrich (St. Louis, US) and used at the same concentrations. FK506 from LKT Laboratories Inc. (St. Paul, US) was used in phBECs up to 40 µM and Huh-7.5 and HEK293 cells up to 50 µM. The non-immunosuppressive FK506 analogs (compound 16j from (Pomplun et al., 2018) and its more potent analog compound 19^(S)-Me^ from (Bauder et al., 2021)) were used at 10 and 20 µM in all cell lines. The structural formulas of FK506 and its non-immunosuppressive analogs are depicted in **Supplementary Figure 1**. All compounds were diluted in dimethyl sulfoxide (DMSO).

### 2.3 Cell culture

#### 2.3.1 Primary human bronchial epithelial cells

phBECs from three different donors were cultured as described previously (Schamberger et al., 2015; Mastalerz et al., 2022). Cells were first expanded in p2 in PneumaCult™-ExPlus medium (Stemcell Technologies, Vancouver, Canada) supplemented with 1× Supplement, 1% penicillin/streptomycin, and 0.1% hydrocortisone in a 100 mm dish until reaching 85% confluency. They were then seeded onto 12-well transwell insert plates (Corning, New York, US) at a density of 100,000 cells/cm^2^ and allowed to reach confluency, before removing the apical medium to create an air–liquid interface and changing the basolateral medium to PneumaCult™-ALI medium (Stemcell Technologies) supplemented with 10× Complete Base Medium, 1× Maintenance Supplement, 0.2% heparin, 0.5% hydrocortisone, and 1% penicillin/streptomycin. To obtain a fully differentiated bronchial epithelium, cells were allowed to differentiate for 28 days. The apical surface was washed with Hanks’ Balanced Salt Solution (HBSS) on days 7, 14, 21, and 28 of differentiation to remove excess mucus.

#### 2.3.2 Cell lines

The human hepatoma cell line Huh-7.5 (Blight et al., 2002) and the human embryonic kidney cell line HEK293 were cultured in Dulbecco’s modified Eagle medium (Invitrogen, Karlsruhe, Germany) supplemented with 10% fetal bovine serum and 1% penicillin/streptomycin.

#### 2.3.3 Cell viability tests

Cell viability and cytotoxicity were quantified in phBECs using the lactate dehydrogenase release assay (Sigma-Aldrich). Cells were washed on the apical side with pre-warmed 1× HBSS and the supernatant was collected. For each experiment, a high control was prepared by lysing cells in 2% Triton-X in PneumaCult™-ALI medium for maximum LDH release. As a low control, untreated cells were used. Cell-free HBSS was used as a background. The absorbance of the samples was then measured using a plate reader (CLARIOstar Plus, BMG Labtech, Ortenberg, Germany) at 492 nm, and the cytotoxicity was calculated in percent according to the instructions of the manufacturer.

For Huh-7.5 and HEK293 cells, cytotoxicity was measured using the Cell CellTiter-Glo^®^ Luminescent Cell Viability Assay (Promega, Madison, US) according to the manufacturer’s instructions. Briefly, cells were lysed in the kit’s reagent and the luminescent signal of ATP released from metabolically active cells was read by a luminometer (CLARIOstar Plus). Cell free medium was used as a background, and untreated cells were used as a low control.

### 2.4 Pretreatment

Fully differentiated phBECs were pretreated with the compounds at the indicated concentrations. Initially, 20mM stock solutions for all compounds were prepared in DMSO and then diluted in PneumaCult™-ALI medium, not exceeding a final DMSO concentration of 0.1%, a non-cytotoxic DMSO concentration for phBECs under these conditions.

### 2.5 Infection/transfection in the absence and presence of immunophilin inhibitors

For the infection of phBECs, cells were washed twice with 500 µl of HBSS, apically to remove excess mucus. HCoV-229E wild-type virus diluted in serum-free Dulbecco’s modified Eagle medium was used for the infection for 2 h on the apical side of the transwell inserts with an MOI of 4. After infection, the virus was aspirated and the cells were washed twice in 500 µl of HBSS, collecting the second wash for RNA analysis. Supplemented PneumaCult™-ALI medium with inhibitors was added and the cells were left to incubate for 72 h, changing the medium (with inhibitors) after 48 h. After 72 h of incubation, cells were washed with 350 µl of HBSS and the apical supernatant was collected for RNA analysis and cytotoxicity measurements using the LDH assay. TEER measurements were taken to assess barrier integrity (described in Supplementary Materials). Cells were harvested for intracellular RNA analysis with the Isolate II RNA Mini Kit (BioCat GmbH, Heidelberg, Germany) according to the instructions of the manufacturer and stored at −80°C until use. Protein was extracted by lysing the cells in 100 µl of 50 mM Tris–HCl, pH 7.5, 150 mM NaCl, 10 mM DTT, 1% NP-40, and protease inhibitor cocktail (Roche, Basel, Switzerland), left to incubate on ice for 30 min, and centrifuged at 4°C at 14,000 RPM for 15 min. The pellet was discarded and the protein was stored at −20°C until further use.

For the infection of Huh-7.5, we used *Renilla* luciferase-expressing HCoV-229E-RLuc at an MOI of 0.1, largely as described previously (Ma-Lauer et al., 2020). The cells were cultured to 70%–80% confluency followed by infection for 2 h at 37°C, 5% CO_2_. The virus was removed and medium with inhibitors at the indicated concentrations was added. The infected cells were left in incubation for 48 h before readout. *Renilla* luciferase activity was recorded as a measure of viral replication in a luminometer (CLARIOstar Plus).

For studying inhibition of coronavirus replication in HEK293 cells, a SARS-CoV-1 replicon (Kusov et al., 2015) carrying the *Renilla* luciferase gene (SARS-CoV-1-RLuc) was transfected into HEK293 cells using Lipofectamine 3000 Reagent (Thermofisher Scientific/Invitrogen) and again recorded the luminescent signal as a measure of viral replication after 48 h.

## 3. Results

### 3.1 Cyclosporin A and its non-immunosuppressive analog Alisporivir inhibit coronavirus replication largely independent of cell type and virus strain

**Figure 1:**
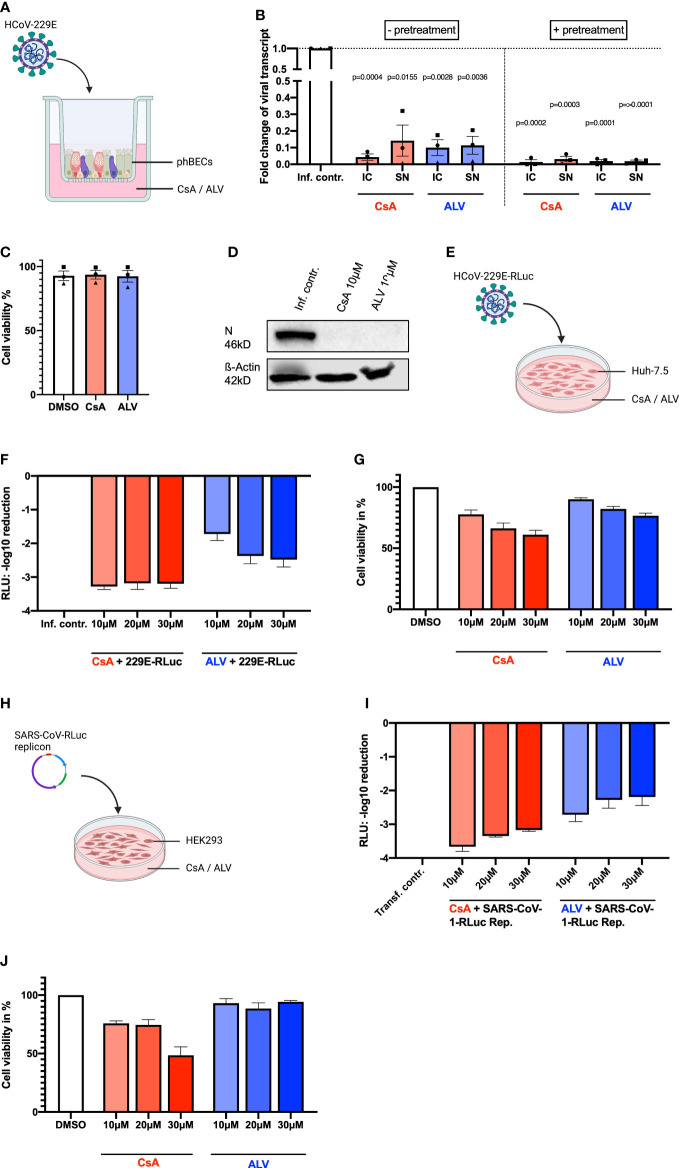
Inhibitory effects of CsA and ALV in different human infection models. **(A)** Illustration of phBEC infection with HCoV-229E, created with biorender.com. **(B)** RT-qPCR results of (for 72 h) HCoV-229E infected (MOI = 4) phBECs (n = 3, independent donors) with and without pretreatment in presence of compound CsA and ALV at 10 µM, given as fold changes of intracellular (IC) and supernatant (SN) viral transcript relative to the infection control (inf. contr.), HCoV-229E-infected phBECs treated with the vehicle DMSO (normalized to 1 for both IC and SN). Intracellular: Normalized to the housekeeping gene DEAH-Box Helicase 8 (*DHX8*). The symbols each represent an independent donor (circles = donor 1; squares = donor 2; triangles = donor 3). For statistical analysis, a paired two-tailed t-test was used and absolute *p*-values are given for *p <*0.05. **(C)** Cell viability was assessed in the absence of virus by the LDH assay after 48 h pretreatment plus 72 h mock infection in percent. Both mock-infected and coronavirus-infected cells had been pretreated with 10 µM inhibitor in medium for 48 h until infection. Following infection the basolateral medium was fully removed and fresh medium with the same inhibitor concentration (i.e., 10 µM) added, keeping the concentration constant at any given timepoint. **(D)** Representative immunoblot analysis of HCoV-229E N protein and loading control β-actin. **(E)** Illustration of Huh-7.5 infected with a variant of HCoV-229E expressing *Renilla* luciferase. Figure was created with biorender.com. **(F)** HCoV-229E infection of Huh-7.5 cells for 48 h: *Renilla* luminescence was quantified as a measure of viral replication. The results are shown in log scale (log_10_ reduction). **(G)** Cell viability of Huh-7.5 cells after 48 h of inhibitor treatment was measured using the Celltiter-Glo assay and is shown in percent. **(H)** Illustration of transfection of HEK293 cells with a pBAC-SARS-Rep-Rluc plasmid expressing *Renilla* luciferase. Figure was created with biorender.com. **(I)** pBAC-SARS-Rep-Rluc transfection into HEK293 cells for 48 h: *Renilla* luminescence was quantified as a measure of viral replication. The results are shown in log scale (log_10_ reduction). **(J)** Cell viability of HEK293 cells after 48 h of inhibitor treatment measured with the Celltiter-Glo assay. All results are shown as mean ± SEM.

Fully differentiated phBECs from three independent donors were treated with CsA and ALV either prior to and immediately after infection (with pretreatment) or only immediately after infection (w/o pretreatment) with HCoV-229E (**Figure 1A**). Both CsA and ALV significantly inhibited viral replication as assessed by intra- and extracellular viral transcript fold changes (**Figure 1B**). The inhibition of viral replication was more pronounced for pretreated phBECs. The inhibitors were used at non-toxic concentrations (**Figure 1C**). The viral nucleocapsid N is a multifunctional protein, which plays an essential role during viral self-assembly (McBride et al., 2014; Chang et al., 2014; Bai et al., 2021) and production, and its expression serves as a measure of active replication. Assessment of N protein expression by immunoblot analysis confirmed strong inhibition of virus replication in pretreated and HCoV229E-infected phBECs (**Figure 1D**). Next, we infected the liver hepatoma cell line Huh-7.5 with a recombinant HCoV-229E virus expressing *Renilla* luciferase (229E-RLuc) and monitored the cytotoxicity and inhibitory effects of CsA and ALV (**Figure 1E**). Both CsA and ALV drastically decreased *Renilla* luciferase activity, a measure of viral replication in this system, already at a concentration of 10 µM (**Figure 1F**). At the same time, both compounds showed a little dose-dependent cytotoxicity, which was more pronounced for CsA, but weak for both compounds at a concentration of 10 µM (**Figure 1G**). Finally, we transfected the human embryonic kidney cell line HEK293 with a SARS-CoV-1 replicon (Kusov et al., 2015) harboring the gene for *Renilla* luciferase (**Figure 1H**), and subsequently treated the cells with CsA and ALV. Again, both compounds drastically reduced *Renilla* luciferase activity (**Figure 1I**). Very similar to our observations in Huh 7.5 cells, CsA displayed moderate, dose-dependent cytotoxicity, while there was negligible cytotoxicity for ALV (**Figure 1J**).

### 3.2 CsA and ALV deplete cyclophilin B at protein level in phBECs

**Figure 2:**
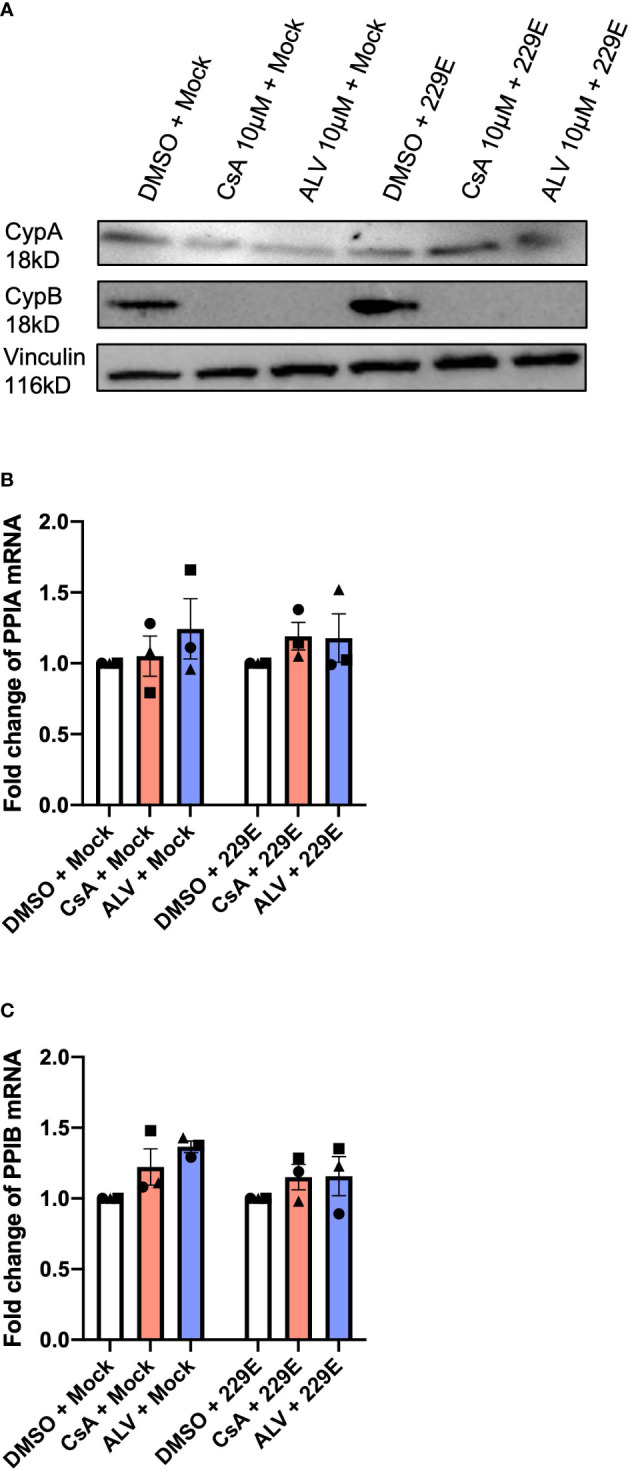
CsA and ALV strongly downregulate CypB but not CypA protein levels in phBECs. **(A)** Immunoblot analysis of total cell lysate after CsA and ALV treatment reveals strong downregulation of CypB protein levels as opposed to comparably little effect on CypA, both in presence and absence of infection. Blot is representative of n = 3 independent experiments **(B)** RT-qPCR of *PPIA* expression depicted as fold changes normalized to the housekeeping gene DEAH-Box Helicase 8 (*DHX8*). The symbols each represent an independent donor (circles = donor 1; squares = donor 2; triangles = donor 3). **(C)** RT-qPCR of *PPIB* expression depicted as fold changes normalized to the housekeeping gene DEAH-Box Helicase 8 (*DHX8*). The symbols each represent an independent donor (circles = donor 1; squares = donor 2; triangles = donor 3). The drug and infection treatment of phBECs were the same in the RT-qPCR as well as the immunoblot analysis (48 h pretreatment, followed by 72 h HCoV-229E infection).

In an effort to gain further insight into the mechanism by which CsA and ALV inhibit coronavirus replication, we performed RT-qPCR and immunoblot analysis to assess the gene expression of the cyclophilins *PPIA* (CypA) and *PPIB* (CypB). CsA and ALV both drastically depleted CypB on protein level in both HCoV-229E infected and mock-infected samples. Effects for CypA protein were much less pronounced and more variable: levels were also downregulated in mock-infected samples, and slightly upregulated in HCoV-229E infected samples (**Figure 2A**). In contrast, RT-qPCR results showed no effect of CsA and ALV on *PPIA* or *PPIB* mRNA levels (**Figures 2B, C**), suggesting post-transcriptional modification of CypA and CypB.

### 3.3 FK506 inhibits coronavirus replication in Huh-7.5 and HEK293, but not consistently in phBECs

**Figure 3:**
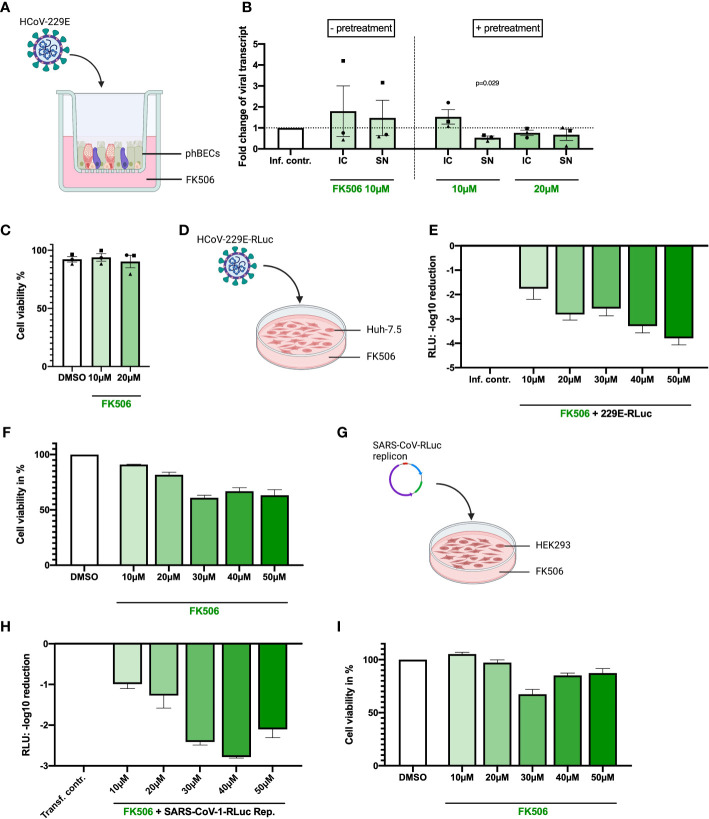
Inhibitory effects of FK506 in different human infection models. **(A)** Illustration of phBEC infection with HCoV-229E, created with biorender.com. **(B)** RT-qPCR results of (for 72 h) HCoV-229E infected (MOI = 4) phBECs (n = 3, independent donors) with and without pretreatment in presence of compound FK506, given as fold changes of intracellular (IC) and supernatant (SN) viral transcript relative to the infection control (inf. contr.), HCoV-229E-infected phBECs treated with the vehicle DMSO (normalized to 1 for both IC and SN). Intracellular: Normalized to the housekeeping gene DEAH-Box Helicase 8 (*DHX8*). The symbols each represent an independent donor (circles = donor 1; squares = donor 2; triangles = donor 3). For statistical analysis a paired two-tailed t-test was used and absolute *p*-values are given for *p <*0.05. **(C)** Cell viability was assessed by LDH assay after drug treatment for 48 h pretreatment followed by 72 h post infection in percent. Both mock-infected and coronavirus-infected cells had been pretreated with 10 µM inhibitor in medium for 48 h until infection. Following infection the basolateral medium was fully removed and fresh medium with the same inhibitor concentration (i.e., 10 µM) added, keeping the concentration constant at any given timepoint. **(D)** Illustration of Huh-7.5 infected with a variant of HCoV-229E expressing *Renilla* luciferase. Figure was created with biorender.com. **(E)** HCoV-229E infection of Huh-7.5 cells for 48h: *Renilla* luminescence was quantified as a measure of viral replication. The results are shown in log scale (log_10_ reduction). **(F)** Cell viability of Huh-7.5 cells was measured after 48 h of inhibitor treatment using the Celltiter-Glo assay and is shown in percent. **(G)** Illustration of transfection of HEK293 cells with a pBAC-SARS-Rep-Rluc plasmid expressing *Renilla* luciferase. Figure was created with biorender.com. **(H)** pBAC-SARS-Rep-Rluc transfection into HEK293 cells for 48 h: *Renilla* luminescence was quantified as a measure of viral replication. The results are shown in log scale (log_10_ reduction). **(I)** Cell viability of HEK293 cells was measured after 48 h of inhibitor treatment with the Celltiter-Glo assay. All results are shown as mean ± SEM.

The immunophilin inhibitor FK506 has been reported to inhibit HCoV-229E, -NL63, and SARS-CoV-1 replication in Huh-7.5 cells (Carbajo-Lozoya et al., 2012) but had not been tested in phBECs. Hence, equivalent to our experiments with CsA and ALV reported above, fully differentiated phBECs from three independent donors were treated with FK506 either prior to infection (with pretreatment) or only immediately after infection (w/o pretreatment) with HCoV-229E (**Figure 3A**). At non-toxic concentrations of 10 and 20 µM, FK506 had very little to no effect on viral replication in phBECs (**Figures 3B, C**). As this was in contrast to previous reports in other cell lines, which demonstrated clear inhibitory effects on viral replication (Pfefferle et al., 2011; Carbajo-Lozoya et al., 2012), we carried out additional experiments in phBECs derived from two independent donors, where we increased FK506 concentrations up to 40 µM and included pretreatment to maximize potential effects (**Supplementary Figure 2**). Interestingly, while again, at a concentration of 10 µM, we observed no inhibition of coronavirus replication, increasing concentrations revealed some donor-specificity: In phBECs derived from one donor (depicted as black squares in **Supplementary Figures 2B, C**), FK506 showed cytotoxicity from 30 µM, with little to no inhibitory effect. At 40 µM, reduction of viral replication was observed in this donor, which, however, was associated with compound cytotoxicity and thus likely no real inhibitory effect. In phBECs derived from a different donor (depicted as red triangles**)**, however, FK506 inhibited viral replication at non-toxic concentrations higher than 10 µM. The inhibitory effect in this donor was also confirmed by loss of intracellular viral N protein upon FK506 treatment (**Supplementary Figure 2D**).

In striking contrast to our observations in phBECs, non-cytotoxic concentrations of FK506, including 10 µM, consistently inhibited HCoV-229E replication in Huh-7.5 cells (**Figures 3E, F**). The same effect, albeit slightly less prominent, was observed for SARS-CoV-linked luminescence in HEK293-transfected cells (**Figures 3H, I**). In both cell lines, cell viability started to decrease considerably at 30 µM.

### 3.4 The non-immunosuppressive FK506 analogs 16j and 19^(S)-Me^ do not inhibit coronavirus replication

**Figure 4:**
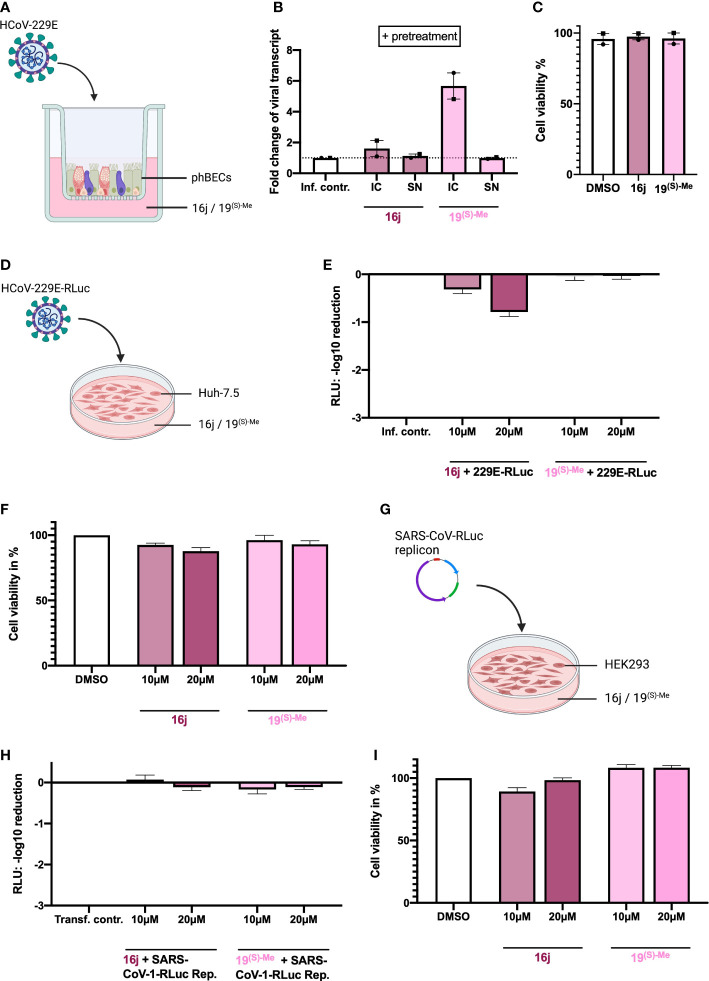
Inhibitory effects of non-immunosuppressive FK506 analogs in different human infection models. **(A)** Illustration of phBEC infection with HCoV-229E, created with biorender.com. **(B)** RT-qPCR results of (for 72 h) HCoV-229E infected (MOI = 4) phBECs (n = 2, independent donors) with pretreatment in presence of compounds 16j and 19^(S)-Me^ (used at 10 µM), given as fold changes of intracellular (IC) and supernatant (SN) viral transcript relative to the infection control (inf. contr.), HCoV-229E-infected phBECs treated with the vehicle DMSO (normalized to 1 for both IC and SN). Intracellular: Normalized to the housekeeping gene DEAH-Box Helicase 8 (*DHX8*). The symbols each represent an independent donor (circles = donor 1; squares = donor 2). **(C)** Cell viability as assessed by LDH assay after 48 h pretreatment and 72 h post infection in percent. Both mock-infected and coronavirus-infected cells had been pretreated with 10 µM inhibitor in medium for 48h until infection. Following infection the basolateral medium was fully removed and fresh medium with the same inhibitor concentration (i.e., 10 µM) added, keeping the concentration constant at any given timepoint. **(D)** Illustration of Huh-7.5 infected with a variant of HCoV-229E expressing *Renilla* luciferase. Figure was created with biorender.com. **(E)** HCoV-229E infection of Huh-7.5 cells for 48 h: *Renilla* luminescence was quantified as a measure of viral replication. The results are shown in log scale (log_10_ reduction). **(F)** Cell viability of Huh-7.5 cells was measured after 48 h of inhibitor treatment using the Celltiter-Glo assay and is shown in percent. **(G)** Illustration of transfection of HEK293 cells with a pBAC-SARS-Rep-Rluc plasmid expressing *Renilla* luciferase. Figure was created with biorender.com. **(H)** pBAC-SARS-Rep-Rluc transfection into HEK293 cells for 48 h: *Renilla* luminescence was quantified as a measure of viral replication. The results are shown in log scale (log_10_ reduction). **(I)** Cell viability of HEK293 cells was measured after 48 h of inhibitor treatment with the Celltiter-Glo assay. All results are shown as mean ± SEM.

Having observed cell type-specific effects of FK506, we additionally set out to examine non-immunosuppressive FK506 analogs (16j (Pomplun et al., 2018) and 19^(S)-Me^ (Bauder et al., 2021) as potential inhibitors of coronavirus replication in this context. Here, we did not observe any inhibitory effects of the analogs at non-toxic concentrations up to 20 µM, either in phBECs or in Huh-7.5 nor in HEK293 cells (**Figure 4**).

## 4 Discussion

Previous studies largely relying on non-airway-derived cancer cell lines have put forward immunophilin inhibitors, including CsA and FK506, as inhibitors of coronavirus replication. In this study, we assessed the anti-coronaviral potential of CsA, FK506, and non-immunosuppressive analogs of both in an organotypic model of the bronchial epithelium as well as in other frequently used human models of coronavirus infection. Our findings suggest that the cyclophilin inhibitors CsA and ALV inhibit coronavirus replication largely independent of virus strain and infection model at non-toxic low-micromolar concentrations. Furthermore, pretreatment with the respective inhibitors augmented their inhibitory effects in bronchial epithelial cells. In contrast, FK506 displayed cell type-specific effects, inhibiting coronavirus replication in Huh-7.5 and HEK293 cells but only poorly in phBECs.

Immunophilin inhibitors are clinically used in transplant medicine to prevent allograft rejection. Their immunosuppressive properties rely on their ability to form a complex with CypA or FKBP12, which in turn binds to the phosphatase calcineurin and inhibits its activity (de Wilde et al., 2018). In principle, CsA and FK506 act as adapters between CypA and FKBP12, respectively, on one side, and calcineurin on the other (Huai et al., 2002). Inhibition of calcineurin blocks the nuclear factor of the activated T-cells (NFAT) signaling pathway and subsequently inhibits IL-2 production and T-cell activation. Hence, both immunophilin inhibitors may blunt the normal antiviral T-cell response and thus ultimately promote virus replication. Therefore, in this study, we included non-immunosuppressive analogs of both compounds, which do not have a functional calcineurin-binding domain. ALV, a non-immunosuppressive analog of CsA, was similarly effective as CsA in all infection models used, indicating that the structural component important for immunosuppression is not required for coronavirus inhibition and suggesting that ALV is more broadly applicable as an anti-viral agent than CsA. In contrast, while FK506 demonstrated considerable inhibitory potential in two human coronavirus infection models, the two non-immunosuppressive analogs did not inhibit coronavirus replication in any human infection model assessed. These inhibitors are pan-selective, highly efficient ligands of human FKBPs with a high structural similarity to FK506 except for their immunosuppressive site, the calcineurin binding domain, which is substituted (Pomplun et al., 2018). 16j (Ziegler et al., 2020) and 19 (S)-Me (Pomplun et al., 2018) are both highly cell permeable (Gnatzy et al., 2021), excluding limited cell permeability as a reason for the lack of efficacy for the non-immunosuppressive FK506. Therefore, the lack of inhibitory effect of 16j (Pomplun et al., 2018) and 19^(S)-Me^ (Bauder et al., 2021) in all infection models may indicate a more important role of the FK506 calcineurin binding domain in the inhibition of coronavirus replication than for CsA.

Interestingly, while immunocompromised patients, including those undergoing immunosuppressive therapy after solid organ transplantation, are listed as high-risk patients for severe COVID-19, observational studies have not provided evidence that CsA treatment may increase susceptibility to SARS-CoV-2 infection or lead to a more severe outcome (Devaux et al., 2021). In contrast, some clinical studies have even raised the intriguing possibility that calcineurin inhibitors may be beneficial for outcomes (Poulsen et al., 2020). For instance, a preliminary single-center observational study with 29 kidney transplant recipients infected with SARS-CoV-2, indicated that CsA may lead to lower mortality (Rodriguez-Cubillo et al., 2020). An independent multicenter study with 40 kidney-transplanted patients also found that CsA significantly reduced mortality (Demir et al., 2020). Most strikingly, another observational single-center study (607 patients), where the impact of multiple prescribed therapies on clinical outcome after COVID-19 was assessed, identified CsA as the only therapy that was associated with a significant decrease in mortality (Guisado-Vasco et al., 2020). Likewise, in a study involving 243 liver transplant recipients, continuous use of FK506 was associated with improved patient survival (Belli et al., 2021; Yin et al., 2021). Overall, these findings indicate that calcineurin inhibitors do not necessarily exacerbate infection and that they, in particular CsA, may even protect against severe COVID-19. Notably, using pretreatment of phBECs, we have modelled this preventive approach *in vitro* and demonstrated that CsA and ALV are even more effective in inhibiting coronavirus replication in this scenario than when given simultaneously with infection. Hence, direct inhibition of coronavirus replication may contribute to the observed protective effects of CsA in transplant recipients with COVID-19. Intriguingly, in a translational mouse model of SARS-CoV-2 infection, CsA led to an efficient reduction of viral RNA while leaving the total number of T cells in the bronchoalveolar lavage unchanged, suggesting maintained T-cell activation despite systemic CsA treatment (Sauerhering et al., 2022). Clearly, more studies are needed to unravel the reciprocal influence of the antiviral and immunomodulatory activities of CsA and related compounds.

The potent antiviral effects of CsA have also been demonstrated for SARS-CoV-2 infection in several relevant preclinical models, such as phBECs (Sauerhering et al., 2022), primary human nasal epithelial cells (Kratzel et al., 2021), lung donor-derived precision cut lung slices, and the above-mentioned mouse model (Sauerhering et al., 2022). Given the well-established antiviral properties of CsA, it is not surprising that several FDA-approved clinical trials are underway to investigate the effect of CsA on COVID-19 outcomes in more detail (Devaux et al., 2021). However, the molecular mechanism by which CsA and ALV inhibit coronavirus replication remains poorly understood. Here, we observed that CsA and ALV dramatically reduced CypB protein levels in phBECs in both infected and mock-infected samples. This effect has been reported before in other cell lines (Wei et al., 2018; Ma-Lauer et al., 2020). The unchanged *PPIB* mRNA-level points to some form of post-transcriptional mechanism underlying the loss of CypB, e.g., increased proteasomal degradation, the induction of autophagy, or microRNA-based repression of protein translation, possibly by the formation of a CypB/CsA complex. CypB harbors an ER retention signal and is thus an endoplasmic reticulum (ER) resident protein (Derkx and Madrid, 2001; Sarro et al., 2020; Khong et al., 2020). In untreated Huh7 cells (without virus or inhibitor), CypB localizes to the cytoplasm and ER. CsA/ALV treatment strongly reduces CypB expression in both compartments, and a small fraction of the protein translocates to granular structures in the nucleus. In the presence of HCoV-229E (without inhibitor), CypB shifts from the cytoplasm to intense bleb-like structures in the ER (Ma-Lauer et al., 2020).

The expression levels of CypA in Huh7 cells were not affected by the virus or inhibitors. Even if by far not as drastically as CypB, CsA and ALV slightly downregulated CypA protein levels in mock-infected samples. However, HCoV-229E infection slightly increased CypA protein levels and counteracted this effect. Yet, similar to *PPIB*, *PPIA* mRNA levels remained unchanged. These findings suggest that CypA may also be an important coronavirus host factor, the expression of which might be promoted upon infection by mechanisms that remain to be elucidated. Interestingly, expression levels and intracellular distribution of CypA in Huh7 are not affected by the virus or inhibitors (Ma-Lauer et al., 2020).

Yeast-Two-Hybrid protein interaction screenings have identified direct interactions between SARS-CoVs non-structural protein 1 (nsp-1) and immunophilin proteins (among others, CypA and CypB) (Pfefferle et al., 2011), suggesting a plausible link between nsp-1 expression and NFAT activation. Nsp-1 is a virulence factor associated with the suppression of the immune response of the host (Yuan et al., 2021) and, with 84.4% sequence identity, is highly conserved between SARS-CoV and SARS-CoV-2 (Min et al., 2020). The current report warrants further studies on the role of viral proteins and cyclophilins as potential host factors.

Why FK506 displayed cell type-specific effects on coronavirus replication in our study remains unclear. The 15 human FKBPs that have so far been identified are characterized by divergent functions, cell type-specific expression, and highly variable binding affinities to FK506 (Lammers et al., 2010; Staab-Weijnitz et al., 2015; Tong and Jiang, 2015; Preisendorfer et al., 2022). To date, it has not been elucidated which FKBP is responsible for the anti-coronaviral effect of FK506, and it is more than likely that *FKBP* expression differs considerably in the three cell types used. Therefore, a plausible explanation for the cell type-specific effects may be missing expression of the critical FK506-sensitive host factor in phBECs in contrast to Huh-7.5 and HEK293 cells.

Furthermore, the effect of FK506 showed considerable donor variability (**Figure 3**, **Supplementary Figure 2**). Different cell type compositions associated with divergent *FKBP* expression or varying infection efficiency in our human bronchial epithelial cells can be excluded as the cause, because the distribution of all main bronchial epithelial cell types was very similar across donors (**Supplementary Figure 3**) (Boers et al., 1998; Boers et al., 1999; Rogers, 2003). Instead, (epi-)genetic or other unique patient characteristics may underlie this observation, which ultimately demonstrates that FK506 may display antiviral properties in some patients, even if less consistently than CsA and structural analogs thereof.

In conclusion, we showed that CsA and ALV inhibited coronavirus replication in all human infection models assessed, including in the physiologically highly relevant organotypic model of the bronchial epithelium. The latter effect was even more pronounced in a preventive approach, suggesting that direct inhibition of coronavirus replication underlies some of the observed protective effects of CsA in transplant patients with COVID-19. This effect appeared independent of the immunosuppressive effect of CsA, as the non-immunosuppressive analog ALV was similarly effective. In contrast, FK506 showed cell-type specific inhibitory effects, dose-dependently and strongly inhibiting CoV replication in Huh-7.5 and HEK293 cells, but only inconsistently and overall less pronounced in phBECs. Furthermore, the two non-immunosuppressive analogs of FK506 used here did not affect coronavirus replication in any used human infection model.

## Data availability statement

The original contributions presented in the study are all included in the article and the **Supplementary Material**. Further inquiries can be directed to the corresponding authors.

## Ethics Statement

The study was approved by the local ethics committee of the Ludwig-Maximilians University of Munich, Germany (Ethic vote #19-630). Written informed consent was obtained from all study participants.

## Author contributions

EB, AvB, and CS-W designed and conceived the research. AvB and CS-W supervised the project. EB, YM-L, AC, and BvB planned and performed experiments and analyzed data. AH, RH, and JB provided clinical samples and primary cells. FH provided essential chemicals and interpreted results. EB, CS-W, and AvB wrote the manuscript. All authors contributed to the article and approved the submitted version.

## Funding

This work was supported by grants of the “Bundesministerium für Bildung und Forschung” of the German Government (RAPID 01Kl1723C/01KI2006C, to AvB, Ludwig-Maximilians-University Munich), the German Center for Infection Research (DZIF, partner site Munich, project TTU EI 01.806 to AvB), the Helmholtz Association, the German Center for Lung Research (DZL, partner site CPC-M, to CS-W), the German Research Foundation (Deutsche Forschungsgemeinschaft, DFG) within the Research Training Group GRK2338 (to CS-W), and the Federal Institute for Risk Assessment (Bundesinstitut für Risikobewertung, BfR, #1328-570, to CS-W). EB received an MD scholarship within the GRK2338. FH was supported by the Pioneer Fund (CoMaProtInhib) and the LOEWE Exploration grant PaaP. We are grateful to Charles Rice for providing the Huh7.5 cell line.

## Acknowledgments

We gratefully acknowledge the provision of human biomaterial (primary human bronchial epithelial cells) and clinical data from the CPC-M bioArchive and its partners at the Asklepios Biobank Gauting, the LMU Hospital and the Ludwig-Maximilians-Universität München. We thank the patients and their families for their support.

## Conflict of interest

The authors declare that the research was conducted in the absence of any commercial or financial relationships that could be construed as a potential conflict of interest.

## Publisher’s note

All claims expressed in this article are solely those of the authors and do not necessarily represent those of their affiliated organizations, or those of the publisher, the editors and the reviewers. Any product that may be evaluated in this article, or claim that may be made by its manufacturer, is not guaranteed or endorsed by the publisher.
