# Supplementary material for: Effects of immunophilin inhibitors and non-immunosuppressive analogs on coronavirus replication in human infection models
Source: Front Cell Infect Microbiol. 2022 Sep 21;12:958634. doi: 10.3389/fcimb.2022.958634 (PMC9534297; doi:10.3389/fcimb.2022.958634)
Supplement: Supplementary file 1 [file DataSheet_1.pdf]

Supplementary material

Effects of immunophilin inhibitors and non-immunosuppressive derivates on coronavirus replication in human infection models

Emilia J. Berthold[1,2], *Yue Ma-Lauer*[2,3], *Ashesh Chakraborty*[1], *Brigitte von Brunn*[2,3], *Anne Hilgendorff*[1], *Rudolf Hatz*[4], *Jürgen Behr*[5], *Felix Hausch*[6], *Claudia A. Staab-Weijnitz*[1*], *Albrecht von Brunn*[2,3*]

[1] Institute of Lung Health and Immunity and Comprehensive Pneumology Center with the CPC-M bioArchive, Helmholtz-Zentrum München, Munich, Germany; Member of the German Center for Lung Research (DZL)
[2] Max von Pettenkofer Institute, Dep. of Virology, Faculty of Medicine, LMU Munich
[3] German Center for Infection Research (DZIF), Munich site
[4] Thoraxchirurgisches Zentrum, Klinik für Allgemeine, Viszeral-, Transplantations-, Gefäß- und Thoraxchirurgie, Klinikum Großhadern, Ludwig-Maximilians-Universität (LMU), Munich, Germany
[5] Medizinische Klinik und Poliklinik V, Klinikum der Ludwig-Maximilians-Universität (LMU), Munich, Germany, Member of the German Center for Lung Research (DZL)
[6] Department of Chemistry and Biochemistry, Technical University Darmstadt, Germany

[*] Corresponding authors:

Claudia Staab-Weijnitz, Comprehensive Pneumology Center, Ludwig-Maximilians-Universität and Helmholtz Zentrum München, Max-Lebsche-Platz 31, 81377 München, Germany, Tel.: 0049(89)31874681; Fax: 0049(89)31874661; E-Mail: staab-weijnitz@helmholtz-muenchen.de
ORCID-ID: https://orcid.org/0000-0002-1211-7834

Albrecht von Brunn, Max von Pettenkofer Institute, Dep. of Virology, Faculty of Medicine, LMU Munich, Pettenkoferstrasse 9a, 80336 Munich, Germany, Tel.: 0049(89)218072839; E-Mail: vonbrunn@mvp.lmu.de
ORCID-ID: https://orcid.org/0000-0002-7068-9689

1. **Supplementary Methods**

1.1 Transepithelial electrical resistance (TEER) measurement

On day 7, 14, 21 and 28 of differentiation as well as during and after treatment epithelial barrier integrity of the phBECs was monitored by measuring transepithelial electrical resistance (TEER) with a Millicell-ERS-2 volt-ohm-meter (Millipore, Burlington, US) and a STX01 chopstick electrode (Millipore). In detail, 500µL of HBSS were added to the apical compartment of the transwell insert and left to equilibrate for 5-10 minutes. Measurements were performed in technical triplicates per insert, a blank value was subtracted from the mean and the resulting value was multiplied with the well surface area (1.12 cm^2 for 12-well transwell inserts from Corning) yielding Ω x cm^2.

1.2 RNA extraction and RT-qPCR analysis

RNA (from supernatants 0h and 72h post infection and intracellular material) from the phBECs was isolated using the Isolate II RNA Mini Kit (bioline meridian Bioscience) according to manufacturer's instructions. The intracellular mRNA was transcribed into cDNA using reverse transcriptase (Invitrogen, Germany) and random hexamer primers (Applied Biosystems, Waltham, US). Real Time qPCR was performed in a 96 well plate in a Light Cycler96® LC480II (Roche) and LightCycler® 480 DNA SYBR Green I Master (Roche). Data were calculated by the -ΔΔCt method [1] and normalized to the housekeeping gene DEAH-box helicase 8 (*DHX8*), as endogenous control. Supernatants were analyzed using the Probe RT-qPCR system (SensiFAST™ probe Hi-ROX One-Step Kit, bioline meridian Bioscience).

Supplementary Table 1: List of oligonucleotides used for RT-qPCR analysis

Gene	Forward primer Sequence (5'-3')	Reverse primer Sequence (5'-3')	Probe
229E - N [2]	CAGTCAAATGGGC TGATGCA	AAAGGGCTATAAA GAGAATAAGGTAT TCT	CCCTGACGACCAC GTTGTGGTTCA
DHX8	TGACCCAGAGAAG TG GGAGA	ATCTCAAGGTCCT CATCTTCTTCA	
PPIA [3]	TATCTGCACTGCC AAGACTGAGTG	CTTCTTGCTGGTCT TGCCATTCC	
PPIB	CCAAAGTCACCGT CAA	CAAATCCTTTCTCT CCTGTA	

1.3 Protein analysis

Harvested protein of phBECs were separated by gel electrophoresis at 120V for 80 minutes in a 14% Novex Tris-Glycine gel (ThermoFisher Scientific, Waltham, US) and blotted onto nitrocellulose membranes for 1h at 100V. The membranes were blocked in 5% milkpowder in TBS buffer. Afterwards they were incubated with primary antibodies at 4°C overnight. The secondary antibody was applied for 2 hours at room temperature. Between incubation steps membranes were washed with TBS-T buffer.

Supplementary Table 2: List of primary antibodies used for Western Blot analysis

Target	Host	Ref no	Provider	Dilution
Viral N protein	Mouse	1H11	Eurofins Ingenasa (Madrid, Spain)	1:400
CypA	Rabbit	ab3563	Abcam (Cambridge, UK)	1:500
CypB	Rabbit	PA1-027A	ThermoFisher Scientific	1:800
Vinculin	Mouse	V9264	Sigma-Aldrich	1:1000
ß-Actin peroxidase	Mouse	A3854	Sigma-Aldrich	1:50000
Anti-Rabbit	Goat	a120-201p	Bethyl Laboratories (Montgomery, US)	1:1000
Anti-mouse peroxidase	Goat	A9917-1ML	Sigma-Aldrich	1:20000

1.4 Immunofluorescence staining

To ensure full differentiation on day 28 after airlift, transwell membranes containing phBECs were stained with cell type specific markers and the cell type quantification was performed as described previously [4].

1.5 Statistical analysis

The results are shown as mean ± SEM from at least three independent experiments. For statistical analysis, a paired students *t*-test was carried out using the GraphPad Prism 9 Software (GraphPad Software, San Diego, US).

2. Supplementary Figures

Supplementary Figure 1: Structural formulars of FK506 and its non-immunosuppressive analogues

(A) Shows structural formula of FK506. **(B)** Shows structural formula of 16j [5]. **(C)** Shows structural formula of $19^{(S)\text{-}Me}$ [6].

A

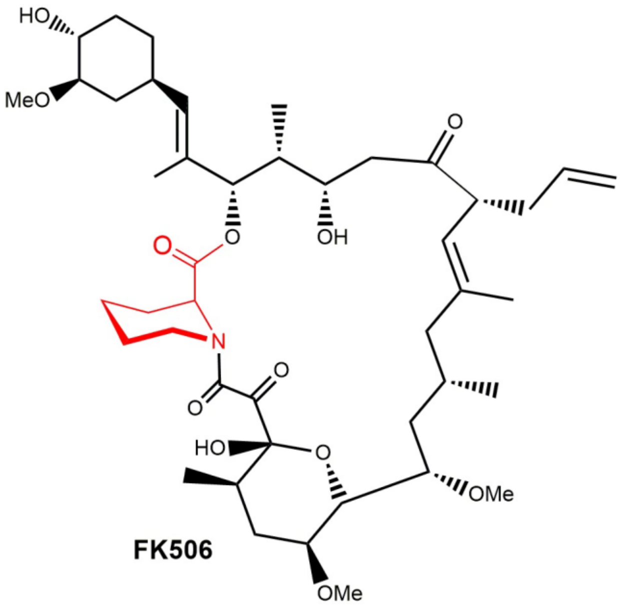

B

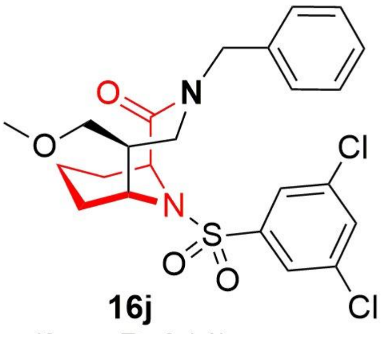

C

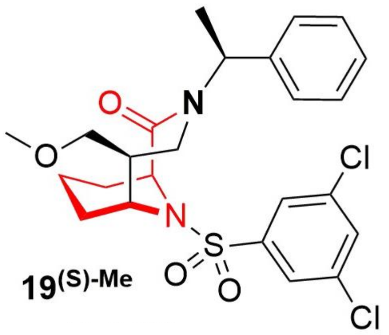

Supplementary Figure 2: Inhibitory effects of FK506 up to 40µM in phBECs. (A) Illustration of phBEC infection with HCoV-229E, created with biorender.com**. (B)** RT-qPCR results of (for 72h) HCoV-229E infected (MOI=4) phBECs (n=2, independent donors) with pretreatment in presence of FK506, given as fold changes of intracellular (IC) and supernatant (SN) viral transcript relative to the infection control treated with the vehicle DMSO. Intracellular: Normalized to the housekeeping gene DEAH-Box Helicase 8 (*DHX8*). The symbols each represent an independent donor (black squares = donor 2; red triangles = donor 3) **(C)** Cell viability was assessed by LDH assay after 48h pretreatment and 72h post infection in %. **(D)** Immunoblot analysis of HCoV-229E N protein of donor 3 and loading control ß-Actin. All results are shown as mean ± SEM.

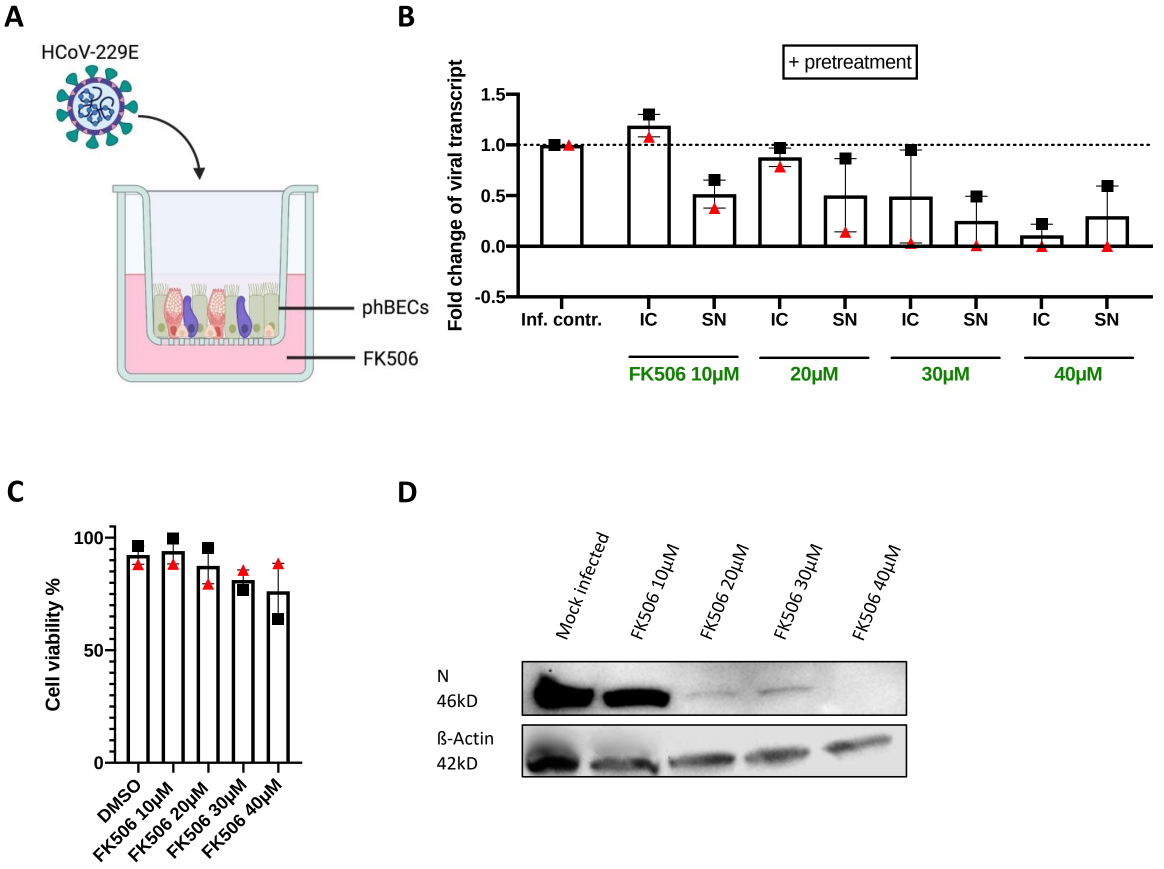

Supplementary Figure 3: PhBECs differentiated for 28 days at ALI represent a full-blown bronchial epithelium with all four major cell types. (A) Immunofluorescent (IF) stainings at day 28 of differentiation of all three phBEC donors used for infection experiments. Four specific markers for the main cell types of the bronchial epithelium were used, namely p63 as a marker of basal cells, mucin 5AC (MUC5AC) for goblet cells, acetylated tubulin (acTub) for ciliated cells, and club cell specific protein (CC10) for club cells. Nuclei were stained with DAPI. (**B)** Cell quantification based on IF staining in % shows similar cell composition in all three donors.

A

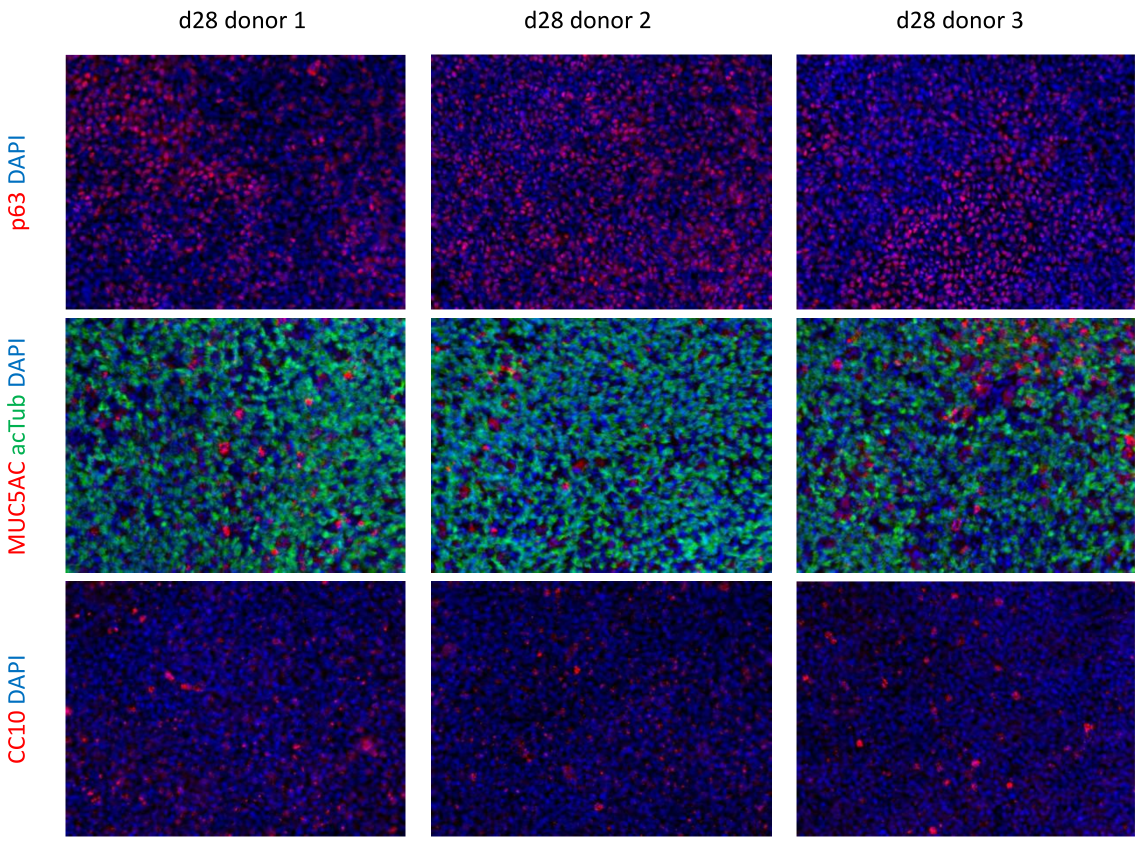

B

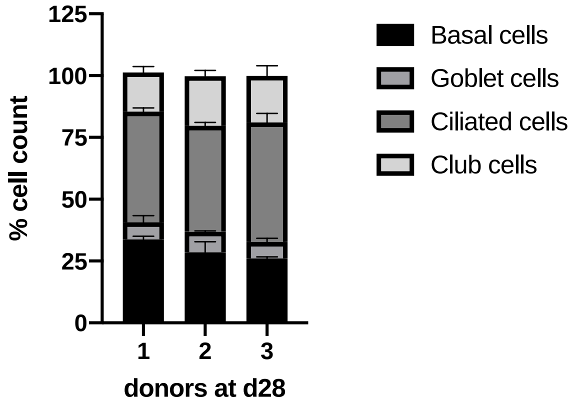

3. Supplementary References

1. Livak, K.J. and T.D. Schmittgen, *Analysis of relative gene expression data using real-time quantitative PCR and the 2(-Delta Delta C(T)) Method.* Methods, 2001. **25**(4): p. 402-8.
2. Zhang, S.F., et al., *Epidemiology characteristics of human coronaviruses in patients with respiratory infection symptoms and phylogenetic analysis of HCoV-OC43 during 2010-2015 in Guangzhou.* PLoS One, 2018. **13**(1): p. e0191789.
3. Batalha, V.L., et al., *The caffeine-binding adenosine A2A receptor induces age-like HPA-axis dysfunction by targeting glucocorticoid receptor function.* Sci Rep, 2016. **6**: p. 31493.
4. Mastalerz, M., et al., *Validation of in vitro models for smoke exposure of primary human bronchial epithelial cells.* Am J Physiol Lung Cell Mol Physiol, 2022. **322**(1): p. L129-L148.
5. Pomplun, S., et al., *Chemogenomic Profiling of Human and Microbial FK506-Binding Proteins.* J Med Chem, 2018. **61**(8): p. 3660-3673.
6. Bauder, M., et al., *Structure-Based Design of High-Affinity Macrocyclic FKBP51 Inhibitors.* J Med Chem, 2021. **64**(6): p. 3320-3349.
